# Comparison of Prelaminar Thickness between Primary Open Angle Glaucoma and Normal Tension Glaucoma Patients

**DOI:** 10.1371/journal.pone.0120634

**Published:** 2015-03-20

**Authors:** Youn Hea Jung, Hae-Young L. Park, Kyoung In Jung, Chan Kee Park

**Affiliations:** Department of Ophthalmology and Visual Science, The Catholic University of Korea, Seoul St. Mary's Hospital, Seoul, Korea; Casey Eye Institute, UNITED STATES

## Abstract

**Main Objective:**

The thinning of prelaminar tissue and prelamina cupping is known to occur by ischemia, as we see in anterior ischemic optic neuropathy. Since normal tension glaucoma (NTG) is thought to be more related to vascular factor than in primary open-angle glaucoma (POAG), we hypothesized that prelamina thinning may occur prominently in NTG patients. This study investigated the difference in prelaminar tissue thickness between patients with POAG and NTG and verified the factors related to prelaminar thinning.

**Methods:**

Complete ophthalmic examination including standard automatic perimetry was performed in all patients. The prelaminar tissue thickness was measured in all patients by performing enhanced depth imaging with a Heidelberg Spectralis Optical Coherence Tomography. The retinal nerve fiber layer and optic nerve head parameters were obtained using the Heidelberg Retina Tomography II and Cirrus Optical Coherence Tomography. Various ocular factors and their relationships with prelaminar thickness were analyzed.

**Results:**

The mean prelaminar tissue thickness was significantly thinner in patients with POAG than in those with NTG. The difference in the prelaminar thickness between patients with POAG and those with NTG was greater in the early field defect group than in the moderate and severe field groups. In multivariate analysis, the mean prelaminar thickness was related to the intraocular pressure, mean deviation, cup-disc ratio, and cup volume.

**Conclusions:**

The prelaminar tissue was thinner in patients with POAG than in patients with NTG, and intraocular pressure had a strong influence on the prelaminar thickness in both POAG and NTG. This may indicate that mechanical compression is the main pathogenic factor in both POAG and NTG.

## Introduction

The prelaminar region, also termed the anterior portion of the lamina cribrosa, comprises bundles of retinal ganglion cell (RGC) axons, astrocytes, capillaries, and extracellular material [1,2]. In patients with glaucoma, the principal site of RGC axonal insult has generally been acknowledged to be the laminar region of the optic nerve head [3–5]. However, there is growing evidence that reversal of optic nerve cupping after an acute reduction of intraocular pressure (IOP) occurs due to changes in both the lamina and prelaminar zone.

Parrish II et al. [6] reported reversal of optic nerve head cupping 5 years after surgical reduction of IOP and suggested the possibility of prelaminar neural tissue thickening. Reis et al. [7] also reported thickening of the prelaminar tissue after lowering the IOP by surgery in patients with POAG. Barrancos et al. [8] studied 28 patients with POAG who underwent nonpenetrating deep sclerectomy and concluded that cupping reversal was mainly due to changes in prelaminar tissue thickness. In addition, Agoumi et al. [9] reported thinning of the prelaminar tissue after acute IOP elevation in patients with glaucoma. Based on these studies, it can be suggested that the prelaminar thickness is influenced by the IOP; it is compressed when the IOP increases and becomes thicker when the IOP decreases.

Other studies have demonstrated the effect of ischemia on the prelaminar region. The prelaminar region comprises neural and connective tissue, both of which can become thinned by ischemia [10]. Henkind et al. [11] described atrophy of the prelaminar tissue in the histological findings of a 67-year-old woman with arterial anterior ischemic optic neuropathy (AAION). Danesh-Meyer et al [12]. compared the optic disc morphology among patients with open-angle glaucoma, arteritic anterior ischemic optic neuropathy, and nonarteritic anterior ischemic optic neuropathy (NAION) and demonstrated that at similar degrees of retinal ganglion cell axon loss, there was more dramatic posterior excavation of the lamina in patients with open-angle glaucoma than in patients with AAION and NAION. Furthermore, patients with AAION who had greater ischemic damage than patients with NAION showed more severe changes in the prelaminar tissue than did patients with NAION. Thus, glaucomatous optic disc cupping is thought to be laminar cupping, whereas disc cupping resulting from ischemia of the optic disc may be prelaminar cupping.

In POAG, the mechanism of optic neuropathy is predominantly associated with the IOP. In NTG, although IOP is still an important factor, other pressure-independent factors such as an increased frequency of migraine headaches, Raynaud’s phenomenon, and sleep apnea have been observed, suggesting a vascular role in the nerve damage of patients with NTG [13]. If there is a greater vascular component in NTG than in POAG, disc cupping in patients with NTG may be characterized by a higher proportion of prelaminar cupping than that in patients with POAG. Therefore, in the present study, we compared the prelaminar tissue thickness using enhanced depth imaging (EDI) spectral domain (SD) Optical Coherence Tomography (OCT) between patients with POAG and those with NTG to verify whether mechanical or ischemic factors influence the prelaminar thickness. In addition, various clinical parameters were investigated to define the factors affecting the prelaminar tissue thickness.

## Materials and Methods

### Subjects

A retrospective medical record review of patients who visited Seoul St. Mary’s Hospital, College of Medicine, Catholic University of Korea, Seoul, Korea, between May and September 2013 was performed. All procedures were carried out in accordance with the Declaration of Helsinki. This study was approved by the institutional review board of Seoul St. Mary’s Hospital. The institutional review board waived the need for a written consent from the participants, because of the retrospective design. Patient information was anonymized and de-identified prior to analysis. When both eyes of the patient were eligible for the study, one eye was randomly selected for inclusion. All participants underwent a comprehensive ophthalmic examination including slit-lamp biomicroscopy, best-corrected visual acuity measurement, Goldmann applanation tonometry, gonioscopy, dilated funduscopic examination, color disc photography, red-free retinal nerve fiber layer photography (VX-10; Kowa Optimed, Tokyo, Japan), ultrasound pachymetry (Tomey Corporation, Nagoya, Japan) to measure central corneal thickness, achromatic automated perimetry using the Swedish Interactive Threshold Algorithm 24–2 (Humphrey Visual Field Analyzer; Carl Zeiss Meditec, Inc., Dublin, CA), axial length measurement using ocular biometry (IOL Master; Carl Zeiss Meditec, Inc., Dublin, CA, USA), Cirrus OCT imaging (Carl Zeiss Meditec, Inc., Dublin, CA, USA), Heidelberg Retina Tomograph II imaging (HRT; Heidelberg Engineering, Heidelberg, Germany), and EDI-OCT (Spectralis; Heidelberg Engineering, Heidelberg, Germany). Experienced ophthalmologists performed the OCT to acquire EDI images. For Cirrus OCT, images that are well focused and well centered without eye motion and with signal strengths greater than 6 were used in the analysis. For HRT, images with standard deviation < 30 μm were accepted.

The inclusion criteria were a best-corrected visual acuity of 20/40 or better with a spherical refraction within ±6 diopters, cylinder correction within ±3 diopters, and axial length of 22 to 25 mm. Patients with at least 2 years of follow-up comprising more than three visual field tests were chosen. Those with a history of previous intraocular surgery or any other ophthalmic disease that could cause visual field defects were excluded.

Both POAG and NTG were defined as the presence of a normal anterior chamber and open angle on slit-lamp and gonioscopic examinations, a glaucomatous optic disc (diffuse or focal thinning of the neuroretinal rim), and an abnormal visual field consistent with glaucoma that were confirmed by at least two reliable visual field examinations. In patients with POAG, untreated IOP was >21 mmHg, whereas in patients with NTG, IOP was ≤21 mmHg without any topical medical treatment. Repeated IOP measurements were performed at least four times per patient on different days at different time points for the diagnosis of NTG. The average of at least five IOP measurements under treatment during the previous 12-month period was used as the mean IOP.

A glaucomatous visual field change was defined as demonstration of the following criteria: (1) a cluster of three or more nonedge points with a probability of <5% on a pattern deviation plot and with one of these points having a probability of <1%; (2) glaucoma hemifield test results outside of the normal limits; or (3) a pattern standard deviation of <5%. Visual field defects had to be repeatable on at least two consecutive tests. Glaucoma was categorized into three subgroups based on the mean deviation (MD) of the visual field according to the modified Hodapp—Anderson—Parrish grading scale as follows [14]: early glaucoma, MD ≥ –6 dB; moderate glaucoma, –6 dB < MD ≤ –12 dB; and severe glaucoma, MD ≤ –12 dB. A reliable visual field test was defined as a false-positive error rate of <15%, a false-negative error rate of <15%, and a fixation loss of <20%.

### Measurement of prelaminar tissue thickness

SD-OCT imaging was performed with the Heidelberg Spectralis OCT (Spectralis software v. 5.1.1.0, Eye Explorer Software 1.6.1.0; Heidelberg Engineering) in the EDI mode after pupillary dilation. The technique has previously been described in detail [14]. The EDI-OCT B-scans around the optic nerve head were obtained using the Heidelberg Spectralis OCT with a 20° retinal window. To center the disc in the 10° × 15° rectangle, an internal nasal fixation light was used while the instrument obtained 512 A-scans of the optic nerve head using a 6-mm-long line at approximately 50 μm intervals. The scan of the optic nerve head resulted in approximately 30 cross-sectional B-scans, of which only those that visualized the prelaminar regions were included. For EDI-OCT images, images with acceptable scans, which require quality score > 15 (range, 0–40), and those with clear fundus image with a good optic disc and scan circle visibility during image acquisition, clearly visible retinal nerve fiber layer (RNFL) without interruptions, and continuous scan pattern with no missing or blank areas.

**Fig 1:**
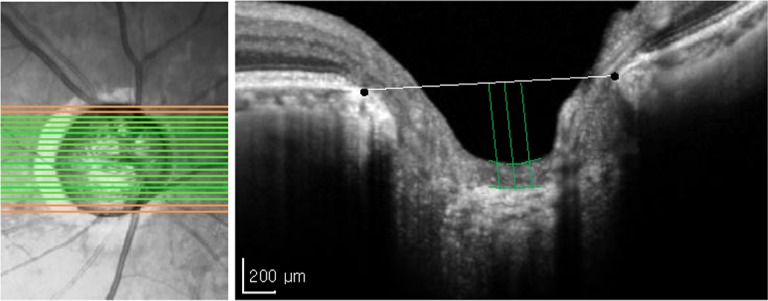
Measurement of the prelaminar thickness. The entire optic nerve head was scanned horizontally with 50-μm intervals. Only those that visualized the prelminar regions (green lines) were included. From a reference line connecting both ends of Bruch’s membrane opening, a perpendicular line was drawn at the center and at 100μm nasally and temporally in each B-scan. For each patient, the mean of 3 prelaminar thickness measurements was defined as the prelaminar thickness of the B-scan. The average of prelaminar thickness of each B-scan was used as the mean prelaminar thickenss

To measure the prelaminar thickness, a perpendicular line was drawn at the center and at 100μm nasally and temporally from a reference line connecting both ends of Bruch’s membrane opening. The prelaminar thickness was measured along this perpendicular line from the anterior border of the reflective region to the anterior border of the highly reflective region, which is the anterior laminar border. For each patient, the mean of 3 values was defined as the prelaminar thickness of the B-scan. The average of prelaminar thickness of each B-scan was used as the mean prelaminar thickenss (Fig. 1).

To evaluate the interobserver reproducibility of our measuring method, 15 randomly selected SD-OCT datasets were evaluated by two independent examiners (YJ and H-YLP), and the intraclass correlation coefficient was calculated. The interobserver intraclass correlation coefficient for measuring the prelaminar tissue thickness was 0.924.

### Statistical analysis

An independent *t* test was performed for continuous variables and Chi-squared test for categorical data to determine the significance of differences between the two groups. When the Levene’s test for equality of variances was statistically significant, indicating the variances were not equal between the two groups, the Welch-Satterthwaite correction was used to account for this. Univariate and multivariate regression analyses were performed to identify factors related to prelaminar thickness. Variables with a significance of *P* < 0.10 in the univariate regression analysis were included in the multivariate regression analysis. The Statistical Package for the Social Sciences for Windows (v. 12.0.0; SPSS Inc., Chicago, IL) was used for all statistical analyses. Values of *P* < 0.05 were considered statistically significant.

## Results

EDI-OCT images were obtained in 176 patients; however, 7 (4.0%) patients (3 POAG and 4 NTG patients) with unclear prelaminar and laminar measurements were excluded from the analysis. A total of 169 patients (68 with POAG and 101 with NTG) were included in the analysis.

### Patients’ baseline characteristics

The patients’ characteristics are summarized in Table 1. There were no statistically significant differences in age, gender, mean intraocular pressure, spherical equivalent, axial length, or central corneal thickness between the POAG and NTG groups. POAG-affected eyes had an average mean deviation of—6.72 dB, which was similar to that of NTG-affected eyes (-6.43 dB, *P* = 0.674). All OCT and HRT parameters, including the RNFL average thickness and vertical cup/disc ratio, were similar between the two groups (Table 2).

**Table 1:** Patient characteristics.

Characteristics	POAG (n = 68)	NTG (n = 101)	*P* value
Age (years)	55.48 (13.47)	57.74 (14.51)	0.360
Gender (Male/Female)	33/35	52/49	0.755
Mean intraocular pressure (mmHg)	14.60 (3.22)	14.00 (2.73)	0.193
Spherical equivalent (D)	-1.27(2.03)	-0.94 (2.17)	0.458
Axial length (mm)	24.13 (1.16)	23.74 (0.75)	0.150
Central corneal thickness (μm)	546.45 (38.00)	536.62 (35.69)	0.423
Mean deviation (dB)	-6.72 (5.73)	-6.43 (5.57)	0.674

POAG = primary open-angle glaucoma; NTG = normal-tension glaucoma.

Data are presented as mean (standard deviation).

Comparisons between the two groups were performed by Student’s *t* test for continuous variables and Chi-squared test for categorical data.

**Table 2:** OCT and HRT parameters of POAG and NTG groups.

Characteristics	POAG (n = 68)	NTG (n = 101)	*P* value
Cirrus OCT			
RNFL average thickness (μm)	74.54 (12.43)	77.84 (12.20)	0.394
Disc area (mm^2^)	2.19 (0.36)	2.16 (0.45)	0.711
Rim area (mm^2^)	0.80 (0.25)	0.85 (0.18)	0.660
Average CDR	0.78 (0.07)	0.76 (0.07)	0.155
Cup volume (mm^3^)	0.65 (0.26)	0.58 (0.28)	0.091
HRT II			
disc size (mm)	2.23 (0.52)	2.22 (0.38)	0.914
Linear CDR	0.71 (0.12)	0.70 (0.08)	0.728
Rim area (mm^2^)	1.05 (0.41)	1.07 (0.27)	0.747
Rim volume (mm^3^)	0.23 (0.15)	0.23 (0.11)	0.917
Mean cup depth (mm)	0.69 (0.15)	0.64 (0.20)	0.207
Cup shape measure	-0.07 (0.08)	-0.07 (0.06)	0.853

OCT = Optical Coherence Tomography; HRT = Heidelberg Retina Tomograph; RNFL = retinal nerve fiber layer; POAG = primary open-angle glaucoma; NTG = normal-tension glaucoma; CDR = cup/disc ratio.

Data are presented as mean (standard deviation).

Comparisons between two groups were performed by Student’s *t* test.

### Prelaminar thickness as measured by EDI SD OCT

**Fig 2:**
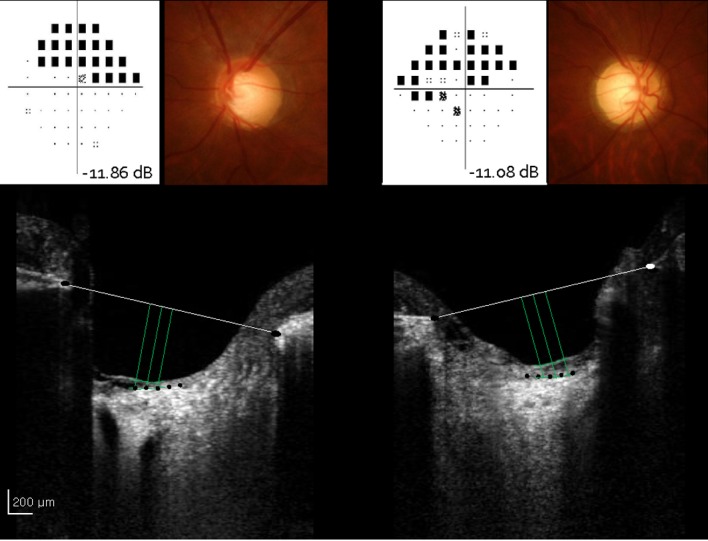
Representative images of patients with POAG and NTG. In POAG-affected eyes, the mean deviation was—11.86 dB, whereas in NTG-affected eyes, the mean deviation was—11.08 dB. The prelaminar tissue was thinner in POAG-affected eyes (75 μm) than in NTG-affected eyes (88 μm). POAG = primary open-angle glaucoma; NTG = normal-tension glaucoma.

The mean (SD) prelaminar thickness was thinner in the POAG group (81.08 (22.50) μm) than in the NTG group (98.35 (32.31) μm) (*P* = 0.000) with statistical significance (Table 3; Fig. 2). The prelaminar thickness was compared between the two groups according to the severity of damage; the mean deviation of the visual field (Table 3). Based on the mean deviation of the visual field, the prelaminar tissue was statistically significantly thinner in the POAG group (89.19 (18.74)μm) than in the NTG group (111.13 (32.92) μm) in the early group (*P* = 0.000). However, there were no statistically significant differences between the POAG and NTG groups in patients with moderate and severe glaucoma (*P* = 0.175 and *P* = 0.311, respectively).

**Table 3:** Mean prelaminar thickness of POAG and NTG groups.

	POAG (n = 68)	NTG (n = 101)	95% Confidence interval of the difference	*P* value
Mean (SD)	81.08 (22.50)	98.35 (32.31)	-25.59 to -8.94	0.000*
By MD of visual field				
Early				
n	26	52		
Mean (SD)	89.19 (18.74)	111.13 (32.92)	-33.62 to—10.26	0.000*
Moderate				
n	22	25		
Mean (SD)	79.18 (22.12)	89.48 (28.25)	-25.35 to 4.76	0.175
Severe				
n	20	24		
Mean (SD)	72.65 (24.75)	79.91 (22.22)	-21.56 to 7.03	0.311

POAG = primary open-angle glaucoma; NTG = normal-tension glaucoma; SD = standard deviation; MD = mean deviation

*Statistically significant value.

In the univariate regression analysis, IOP (*P =* 0.000), visual field MD (*P* = 0.000), average RNFL thickness (*P* = 0.044), rim area (*P* = 0.005), average cup/disc ratio (CDR) (*P* = 0.007), cup volume(*P* < 0.003), linear CDR (*P* = 0.0.007), rim area (*P <* 0.007), and cup shape measure (*P =* 0028) had statistically significant relationships with the prelaminar thickness (Table 4). The multivariate regression analyses included variables with *P* < 0.1 and were performed separately with OCT and HRT parameters due to multicollinearity. In the multivariate regression analysis including OCT parameters, IOP (*P =* 0.001), MD (*P* = 0.001), average CDR (*P* = 0.017), and cup volume (*P =* 0.001), were statistically significant predictors of the prelaminar thickness (Table 5). In the multivariate regression analysis including HRT parameters, IOP (*P* = 0.001) and MD (*P* = 0.007) were statistically significant predictors.

**Table 4:** Univariate regression analysis of prelaminar thickness.

Variable	Univariate analysis		
	Regression coefficient	95% CI	*P* value
Age (years)	0.074	-0.296 to 0.444	0.693
Spherical equivalent (D)	-1.345	-4.199 to 1.508	0.352
Axial length (mm)	5.082	-3.699 to 13.864	0.253
Central corneal thickness (μm)	0.008	-0.112 to 0.129	0.891
Intraocular pressure (mmHg)	-3.074	-4.552 to -1.597	0.000*
Visual field MD (dB)	2.037	0.956 to 3.119	0.000*
Cirrus OCT			
RNFL average thickness (μm)	0.426	0.012 to 0.840	0.044*
Disc area (mm^2^) ^‖^	-0.891	-16.004 to 14.222	0.907
Rim area (mm^2^)	42.774	13.440 to 72.107	0.005*
Average CDR	-114.412	-196.234 to -32.590	0.007*
Cup volume (mm^3^) ^‖^	-32.941	-54.774 to -11.107	0.003*
HRT II			
Disc size (mm)	-0.252	-14.794 to 14.289	0.973
Linear CDR	-85.043	-146.332 to -23.754	0.007*
Rim area (mm^2^)	26.265	7.266 to 45.264	0.007*
Rim volume (mm^3^)	16.591	-32.856 to 66.039	0.507
Mean cup depth (mm)	-21.893	-55.379 to 11.593	0.198
Cup shape measure	-98.156	-185.234 to -11.078	0.028*

MD = mean deviation; OCT = Optical Coherence Tomography; RNFL = retinal nerve fiber layer; CDR = cup/disc ratio; HRT = Heidelberg Retinal Tomography.

* Statistically significant value.

**Table 5:** Multivariate regression analyses of prelaminar thickness.

Variable	Multivariate analysis^†^ ^‡^			Multivariate analysis^†^ ^§^		
	Regression coefficient	95% CI	*P* value	Regression coefficient	95% CI	*P* value
Intraocular pressure (mmHg)	-2.995	-4.785 to -1.205	0.001*	-3.502	-5.455 to -1.548	0.001*
Visual field MD (dB)	2.511	1.272 to 3.75	0.001*	1.55	0.427 to 2.674	0.007*
Cirrus OCT						
RNFL average thickness (μm)	-0.193	-0.823 to 0.437	0.544			
Disc area (mm^2^) ^‖^						
Rim area (mm^2^)	58.038	-21.128 to 137.204	0.149			
Average CDR	223.068	40.726 to 405.410	0.017*			
Cup volume (mm^3^) ^‖^	-142.778	-221.645 to -63.911	0.001*			
HRT II						
Linear CDR				-15.432	-137.816 to 106.954	0.803
Rim area (mm^2^)				24.725	-34.27 to 83.72	0.408
Cup shape measure				-5.258	-129.314 to 118.798	0.933

MD = mean deviation; OCT = Optical Coherence Tomography; RNFL = retinal nerve fiber layer; CDR = cup/disc ratio; HRT = Heidelberg Retinal Tomography.

* Statistically significant value.

† Only variables with *P* < 0.1 in the univariate analysis were included in the multivariate model.

‡ Multivariate analysis including intraocular pressure, visual field MD, and Cirrus OCT parameters with *P* < 0.1 in the univariate analysis.

§ Multivariate analysis including intraocular pressure, visual field MD, and HRT II parameters with *P* < 0.1 in the univariate analysis

‖ For multivariate analyses, squared root of disc area and cubed root of cup volume were included.

### Relationship between IOP and prelaminar tissue thickness

**Fig 3:**
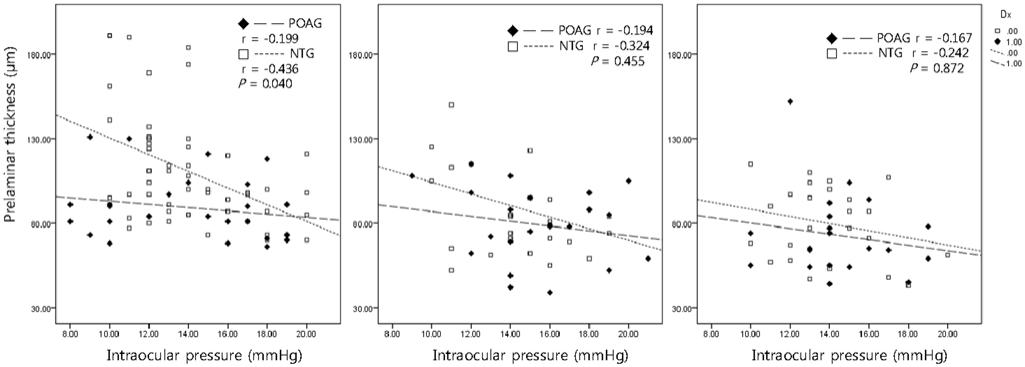
Relationship between IOP and prelaminar thickness in POAG vs NTG patients. There was statistically more negative relationship between IOP and prelaminar thickness in NTG patients than POAG patients in the early glaucoma group (left). In the moderate (middle) and severe (right) groups, the differences were not statistically significant.

The relationship between the IOP and prelaminar tissue thickness in both the POAG and NTG groups was further evaluated by linear regression analysis. The POAG and NTG groups were each subdivided based on the severity of visual field defects. In the mild subgroups, the regression coefficient was more negative in the NTG group (r = -0.436) than the POAG group (r = -0.199) with statistically significance (*P* = 0.040). In the moderate groups, the regression coefficients for NTG and POAG groups were-0.324 and -0.194, respectively with no statistical significance (*P* = 0.455). In the severe groups, there was no statistically significant difference (*P* = 0.872) between regression coefficients in the NTG and POAG groups (r = -0.242 and -0.167, respectively) (Fig. 3).

The NTG group was subdivided according to the prelaminar thickness. The mean (SD) IOP was 14.70 (2.81) mmHg in patients with a prelaminar thickness of <100 μm (n = 60) and 12.97 (2.29) mmHg in those with a prelaminar thickness of ≥100 μm (n = 41); the difference between the two subgroups was statistically significant (*P* = 0.002).

## Discussion

We observed the differences in the prelaminar tissue thickness between patients with POAG and those with NTG using EDI-OCT images and analyzed the factors related to the prelaminar tissue thickness. The prelaminar tissue was significantly thinner in the POAG group than in the NTG group. When we used MD as a surrogate measure of RGC loss, the prelaminar tissue was statistically significantly thinner in the POAG group than in the NTG group within the early MD group. It was also thinner in the POAG group than in the NTG group within the moderate and severe MD groups, but the difference was not statistically significant.

Factors affecting the prelaminar thickness included MD and CDR, indicating that loss of neural tissue resulted in thinning of prelaminar tissue. Disc area and cup volume were also statistically significant factors affecting the prelaminar thickness. Like the laminar tissue, the prelaminar tissue may be influenced by the structural aspects of glaucoma. Furthermore, the IOP had a significant negative relationship with the prelaminar tissue thickness. The prelaminar zone comprises bundles of RGC axons, astrocytes, capillaries, and extracellular material [1,2]. At a similar degree of RGC axon loss, the mechanical force of the IOP may result in a “squeezing” effect, which could explain the difference in the prelaminar thickness between the two groups. Because the prelamina is also more compliant in the early stage of glaucoma, this effect may be more prominent in the early stage of glaucoma, as shown in our results. To the best of our knowledge, this is the first study to compare the difference in prelaminar tissue thickness between patients with POAG and those with NTG. Other studies have shown a relationship between the IOP and prelaminar tissue thickness. Reis et al. [7] reported increased prelaminar tissue thickness after glaucoma surgery in patients with POAG. They speculated that the increase in prelaminar thickness was due to an increase in blood volume or a shift of axoplasmic fluid from the peripapillary retinal nerve fibers or lamina. Lee et al. [15] reported increased prelaminar tissue thickness after trabeculectomy, which decreased the average IOP from 27.2 ± 8.9 to 10.5 ± 3.4 mmHg. However, these studies reported changes in prelaminar tissue thickness after an acute IOP change. Our study demonstrated the chronic effect of IOP on prelaminar tissue thickness and identified other factors that influence the thickness.

The difference in the negative relationship of the IOP and prelaminar tissue thickness between the POAG and NTG groups was the greatest in the early MD group. A previous study by Agoumi et al. [9] compared the prelaminar tissue between patients with glaucoma and controls *in vivo* after an acute IOP elevation. In their study, although their analysis focused on prelaminar tissue displacement, the prelaminar tissue thickness after an acute IOP elevation of approximately 13 mmHg in the patients with glaucoma decreased by 7.3 μm, whereas in age-matched normal controls, the thickness decreased by 20.6 μm. They explained that this decreased compliance in patients with glaucoma may have been due to tissue remodeling of the prelamina, which resulted in increased stiffness. Similarly, an explanation for our findings may be that chronically elevated IOP subjects the prelaminar tissue to consistent stress, which leads to tissue remodeling in the extracellular matrix [16–18]. In patients with advanced glaucoma, not only is there a decrease in RGC axons, but there is also increased stiffness from tissue remodeling, which results in decreased compliance [9].

The prelaminar region comprises neural tissue and connective tissue, which may become thinned by ischemia [10]. Patients with AAION who had greater ischemic damage than that of patients with NAION showed more severe changes in their prelaminar tissue than did patients with NAION at similar degrees of RGC axon loss [12]. Many reports have shown greater vascular roles in NTG than in POAG [19–21]. We hypothesized that if NTG is characterized by a more prominent vascular role than that in POAG, the prelaminar tissue may be more severely affected in NTG eyes. However, the result was the opposite. The prelaminar thickness had a negative correlation with the IOP in both NTG and POAG. This shows that the prelaminar tissue is greatly influenced by the IOP in both POAG and NTG.

Due to the retrospective design, there are only a small number of patients in the subgroups, which requires caution when interpreting these results. To our knowledge, however, this is the first study to compare the prelaminar thickness in POAG and NTG patients, and further studies will be required to assess the difference with different stages of glaucoma.

In conclusion, the prelaminar thickness is influenced by the IOP, MD, CDR, and cup volume in both patients with POAG and those with NTG. POAG-affected eyes demonstrate thinner prelaminar tissue than do NTG-affected eyes at similar levels of glaucoma severity, and the difference is greater in patients with early glaucoma. IOP may be the main pathogenic factor in both POAG and NTG.
